# Solar forcing synchronizes decadal North Atlantic climate variability

**DOI:** 10.1038/ncomms9268

**Published:** 2015-09-15

**Authors:** Rémi Thiéblemont, Katja Matthes, Nour-Eddine Omrani, Kunihiko Kodera, Felicitas Hansen

**Affiliations:** 1 Research Division Ocean Circulation and Climate, GEOMAR Helmholtz Centre for Ocean Research, Düsternbrooker Weg 20, 24105 Kiel, Germany; 2 Christian-Albrechts-Universität zu Kiel, Christian-Albrechts-Platz 4, 24118 Kiel, Germany; 3 Bjerknes Centre and Geophysical Institute, University of Bergen, Postboks 7803, 5020 Bergen, Norway; 4 Solar-Terrestrial Environment Laboratory, Nagoya University, Furo-cho, Chikusa-ku, Nagoya 464-8601, Japan

## Abstract

Quasi-decadal variability in solar irradiance has been suggested to exert a substantial effect on Earth's regional climate. In the North Atlantic sector, the 11-year solar signal has been proposed to project onto a pattern resembling the North Atlantic Oscillation (NAO), with a lag of a few years due to ocean-atmosphere interactions. The solar/NAO relationship is, however, highly misrepresented in climate model simulations with realistic observed forcings. In addition, its detection is particularly complicated since NAO quasi-decadal fluctuations can be intrinsically generated by the coupled ocean-atmosphere system. Here we compare two multi-decadal ocean-atmosphere chemistry-climate simulations with and without solar forcing variability. While the experiment including solar variability simulates a 1–2-year lagged solar/NAO relationship, comparison of both experiments suggests that the 11-year solar cycle synchronizes quasi-decadal NAO variability intrinsic to the model. The synchronization is consistent with the downward propagation of the solar signal from the stratosphere to the surface.

## Abstract (web summary)

While variations in solar irradiance are thought to influence North Atlantic climate variability, the direction of the forcing remains unclear. Here the authors present results from a fully coupled ocean-atmosphere model with interactive chemistry that support a top-down mechanism.

## Graphical abstract

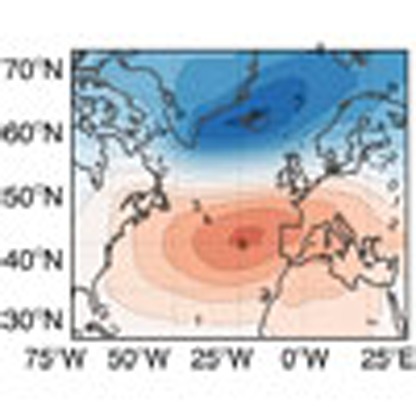

There is increasing evidence that variations in solar irradiance at different time scales are an important source of regional climate variability^1^,^2^,^3^. For instance, observational analyses of sea level pressure (SLP) in winter revealed positive anomalies in the Gulf of Alaska for sunspot peak years since the beginning of the twentieth century^4^,^5^. In the North Atlantic region, a link between the 11-year solar cycle and the winter phase of patterns resembling the North Atlantic oscillation (NAO) or Arctic oscillation (AO) has been found by observational^5^,^6^,^7^,^8^ and modelling^9^,^10^,^11^,^12^ studies. Some of these studies particularly showed that the transfer mechanism leading to the North Atlantic solar signal was consistent with the polar route of the so-called ‘top–down' mechanism^13^ related to solar ultraviolet irradiance variability. According to this mechanism, the solar signal is initially transferred from the upper stratosphere to the lower stratosphere by modulation of the polar night jet and the stratospheric meridional overturning circulation (or Brewer-Dobson circulation^14^), through Rossby wave-mean flow interactions. Dynamical coupling processes^15^ between the stratosphere and the troposphere then transmit the solar signal to the Earth's surface, projecting onto AO/NAO-like patterns^16^.

Recently, analysis of long-term SLP and sea surface temperature observations suggest that the surface climate response to the 11-year solar cycle maximizes with a lag of a few years^17^. These findings were supported by a large ensemble of short-term idealized coupled ocean-atmosphere model experiments^18^, which indicate that the lagged response of the NAO arises from ocean–atmosphere coupling mechanisms. Atmospheric circulation changes associated with the NAO affect the underlying Atlantic Ocean by modulating surface air temperature, atmosphere-ocean heat fluxes, as well as mid-latitude wind stress^19^. This induces a typical sea surface temperature tripolar pattern anomaly that can persist from one winter to the next^20^ and amplify the initial atmospheric solar signal over the subsequent years through positive feedbacks onto the atmosphere^21^,^22^. Although the ocean-atmosphere feedback mechanism has recently been confirmed in an ensemble of transient experiments^23^ of the Hadley Centre Global Environmental Model version 3 (HadGEM3), it remains highly misrepresented by climate models^24^. Furthermore, the detection of the 11-year solar cycle influence on the NAO is arduous since interannual to decadal fluctuations of the latter can be internally generated by the ocean-atmosphere system, even in absence of any external forcing^25^.

Here we present results of two multi-decadal experiments (145 years) of the fully coupled ocean-atmosphere model CESM-WACCM3.5 (ref. 26) in its high-top version (up to 140-km altitude) with interactive chemistry, which either include (SOL experiment) or ignore (NO_SOL experiment) solar variability (see the Methods section). By comparing both experiments, we can analyse the combined role of 11-year solar cycle-induced stratosphere-troposphere couplings and air-sea interactions in setting quasi-decadal fluctuations of the NAO, in a realistic approach. The analysis of the SOL experiment in winter first reveals that our model simulates a lagged and amplified quasi-decadal NAO response to 11-year solar cycle at 1-2 years, consistent with reanalysis. This lagged response simulated in the CESM-WACCM model agrees with the proposed coupled positive feedback between the ocean and atmosphere represented in the HadGEM model^18^,^23^. In the NO_SOL experiment, however, strong internal quasi-decadal fluctuations of the NAO are also found despite constant solar forcing conditions. While our results support that quasi-decadal fluctuations of the NAO can be intrinsically generated in a coupled ocean-atmosphere system^25^, they further suggest that the 11-year solar cycle synchronizes these internal quasi-decadal variations. We show that the proposed synchronization mechanism is consistent with the downward transfer of the solar signal from the stratosphere to the troposphere.

## Results

### North Atlantic climate response to the 11-year solar cycle

**Figure 1:**
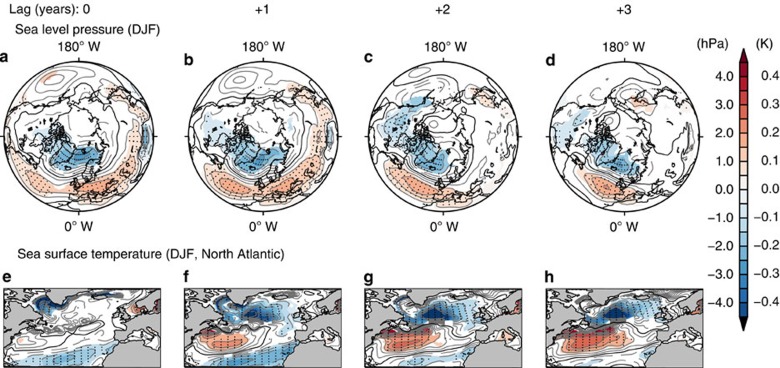
Solar signal at the surface in the Northern Hemisphere. Consecutive lagged composite solar maximum minus minimum differences for sea level pressure (**a**–**d**) and sea surface temperature (**e**–**h**) for DJF season (see the Methods section for details on solar cycle composites definition). Sea level pressure composites are shown for all longitudes and northward of 20° N. Sea surface temperature composites are shown for the North Atlantic region only (80° W–20° E/20° N–70° N). Significance levels are indicated by shaded contours (90%) and dots (95%). DJF, December to February.

In winter (December to February), SLP composite differences between solar maximum and solar minimum phases at lag 0 years (see the Methods section and Supplementary Fig. 1) for the SOL experiment (Fig. 1a) show a statistically significant decrease of 2 hPa over the Arctic region, and a statistically significant increase of 1 hPa in mid-latitudes with maxima in the Euro-Atlantic region and Pacific basin, corresponding to the positive phase of the AO. At lag +1 year (Fig. 1b), the mid-latitude positive SLP anomaly strengthens in the Euro-Atlantic region up to 1.5 hPa and weakens in the Pacific basin resulting in a positive NAO-like pattern. The positive NAO signature still persists at lags +2 and +3 years in the Atlantic basin, but the hemispheric equivalent annular mode signal disappears (Fig. 1c,d). At a lag of +3 years, the statistically significant high-pressure anomaly in mid-latitudes is further amplified, consistent with the ocean feedback mechanism that predicts a maximum response at a lag of +3 years^18^. The corresponding sea surface temperature anomalies (Fig. 1e–h) evolve coherently with the lagged SLP NAO-like response to solar variability. Together with a cold tongue in the southern North Atlantic, a cold anomaly develops in the Labrador Sea (southwest of Greenland) at lag 0 years (Fig. 1e) before intensifying and extending eastwards into the Atlantic at lags +1 and +2 years (Fig. 1f,g). Simultaneously, a warm anomaly strengthens and extends progressively eastward from the middle West Atlantic. As mentioned before, the sea surface temperature cold–warm–cold tripolar pattern^19^ is a typical North Atlantic response to the positive phase of the NAO. This sea surface temperature anomaly, which persists and strengthens over consecutive years, provides a positive feedback onto the atmosphere leading to the lagged and amplified NAO response (Supplementary Fig. 2). Although slightly weaker, the NAO response to the 11-year solar cycle simulated in our model is consistent with reanalysis (Supplementary Fig. 3).

### NAO and solar quasi-decadal variability

**Figure 2:**
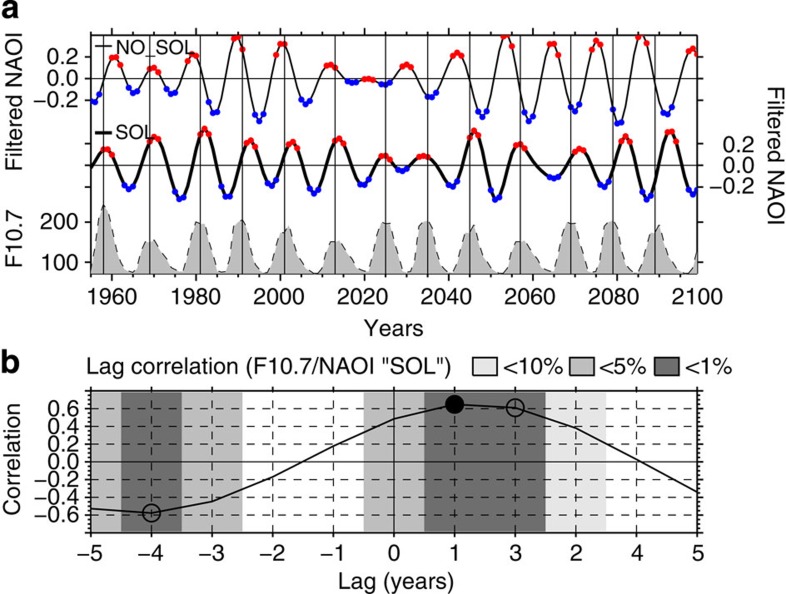
Synchronization of quasi-decadal NAO fluctuations through the 11-year solar cycle. (**a**) Time series of 9–13-year band-pass filtered NAO index for the NO_SOL (solid thin) and SOL (solid thick) experiments, and the F10.7 cm solar radio flux (dashed black). Red and blue dots define the indices used for NAO-based composite differences at lag 0 (see the Methods section). For each solar cycle, maximum are marked by vertical solid lines. (**b**) Lag correlation between F10.7 cm solar radio flux and NAO index of the SOL experiment. Significance levels are indicated by empty (90%) and filled (95%) circles. Grey shades coloured stripes indicate the likelihood (<10, 5 and 1%) that the correlation coefficient between the filtered NO_SOL NAO and the F10.7 time series is higher than between the filtered SOL NAO and the F10.7 ones. All calculations are shown for DJF.

To assess the influence of solar forcing on the NAO temporal variability, we examined wintertime NAO indices of both experiments with regard to the solar radio flux at 10.7 cm (or F10.7 index), which is a measure of solar ultraviolet activity (see the Methods section and Supplementary Fig. 4a–d for details on NAO indices calculation). NAO indices reveal a strong quasi-decadal mode of variability for each experiment as demonstrated by their respective power spectra (Supplementary Fig. 4e,f). This emphasizes that even in the absence of solar variations, an internal quasi-decadal variability mode of the NAO is generated in our model. The comparison of NAO indices spectral properties between the model (Supplementary Fig. 4) and NCEP/NCAR reanalysis (Supplementary Fig. 5) shows peaks, centred at 11 and 9 years, respectively, of similar quasi-decadal power spectral density supporting the realistic nature of the NAO quasi-decadal variability in our model experiments. The quasi-decadal band-pass filtered NAO indices (range 9-13 years; Supplementary Fig. 4e,f) show that for the SOL experiment, the NAO index maxima lag the solar maxima in a coherent manner (Fig. 2a), while for the NO_SOL experiment, the period of this oscillation is not constant over decades. The coherence between the filtered NAO index and the solar variability in the SOL experiment is reinforced by the lagged correlation of both time series, which produces statistically significant correlation coefficients of 0.65 and 0.61 at lag +1 and +2 years, respectively (Fig. 2b). The spectral coherence between the unfiltered SOL NAO and the F10.7 cm time series also indicates a maximum value of 0.72 around a period of 11 years, which is statistically significant at the 95% confidence level (Supplementary Fig. 4e), whereas no significant spectral coherence is found at decadal time scales between the unfiltered NO_SOL NAO and the F10.7 indices (Supplementary Fig. 4f).

In the NO_SOL experiment, the analysis of correlation between the filtered NAO index and the F10.7 revealed that the highest coefficient between lags −5 and +5 years is 0.49 (at lag −3 years) and 0.55 when spanning all possible lags (from −140 to +140 years). Despite the strong NAO quasi-decadal oscillation, the correlation between the filtered NAO index and the F10.7 is thus never as high as for the SOL case. Using a nonparametric random phase method^27^ (see the Methods section for details), we additionally estimated the probability that the correlation coefficient between the filtered NO_SOL NAO and the F10.7 time series is higher than between the filtered SOL NAO and F10.7. We found that this chance is <1% at the lags when maximum positive (+1 and +2) and negative (−4) correlation coefficient are recorded between the filtered SOL NAO and the F10.7 time series (coloured stripes in Fig. 2b). This parallel analysis of the filtered NAO index of both experiments regarding the F10.7 suggests thus that the 11-year solar cycle impacts the North Atlantic climate by synchronizing the internal quasi-decadal variability mode of the NAO simulated by CESM-WACCM (see also Supplementary Fig. 6).

### Stratosphere dynamical analyses

**Figure 3:**
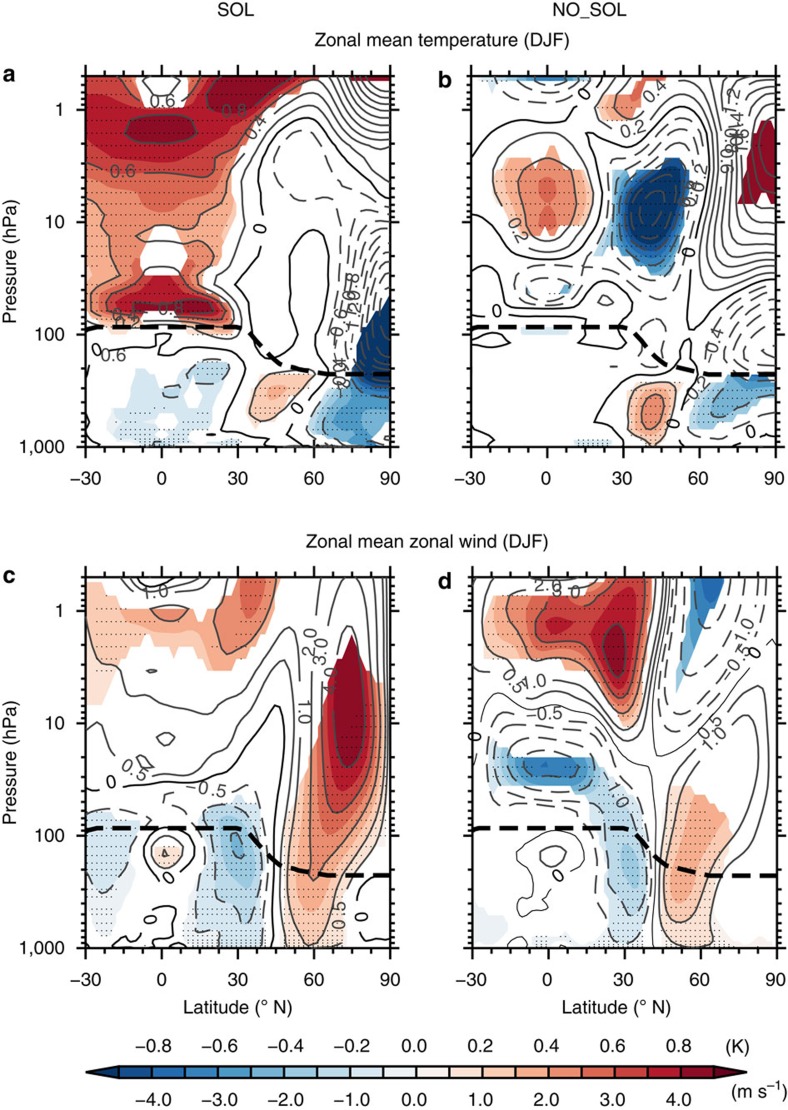
Temperature and zonal wind anomalies associated with NAO quasi-decadal variability and the influence of solar cycle forcing. Latitude-height cross-sections of NAO-based composite differences at lag −1 (corresponding to maximum solar amplitude in the case of the SOL experiment) of the zonal mean temperature (**a**,**b**) and the zonal mean zonal wind (**c**,**d**) for the SOL (**a**,**c**) and the NO_SOL experiment (**b**,**d**). The thick dashed line displays the height of the tropopause. Significance levels are indicated by shaded contours (90%) and dots (95%). All calculations are shown for DJF.

We investigate the proposed synchronization further by comparing the stratospheric response of both experiments with respect to the quasi-decadal NAO. Hence, instead of using 11-year solar cycle composite differences, the analysis that follows is performed by examining the differences between the maximum positive and negative phases of the NAO at quasi-decadal time scales (Fig. 2a; Methods). In the SOL experiment, a statistically significant warming with a maximum of 0.9 K is observed near the tropical stratopause (3–1 hPa≈45–50 km) at lag −1 year of the NAO-based composites (Fig. 3a). This positive temperature anomaly arises from increased solar ultraviolet irradiance during solar cycle maximum phases, which leads to additional heating due to ozone absorption in the upper stratosphere and more ozone production in the mid and upper stratosphere through the photolysis of oxygen^28^. A secondary warming anomaly of 0.8 K appears in the lower equatorial stratosphere (∼70 hPa or 20–25 km). Although less well understood than the direct upper stratospheric response, this secondary warming could arise from adiabatic warming^13^ combined with increased ozone heating^29^ due to a deceleration of the Brewer-Dobson circulation. The two temperature responses to the 11-year solar cycle are in reasonable agreement with reanalyses^30^,^31^. Consistent with the 11-year solar cycle/NAO lagged relationship (Figs 1 and 2), the maximum temperature response in the stratosphere due to 11-year solar variability leads the NAO by 1 year. Conversely, none of these typical solar signals is observed in the NO_SOL experiment which instead shows a seesaw of positive (negative) temperature anomalies in the middle tropical and subtropical stratosphere (20–3 hPa) in winter (Fig. 3b).

In the stratopause region, the 11-year solar cycle induced increase of the poleward temperature gradient results in a strengthening of the subtropical westerly jet through thermal wind balance^13^. Changes in the zonal background flow alter planetary wave propagation so that the initial westerly wind anomalies amplify and propagate poleward and downward through the winter season^13^. This mechanism leads to a deceleration of the Brewer-Dobson circulation and a strengthening of the stratospheric polar vortex at solar maxima. In the SOL experiment, NAO-based composite differences at lag −1 year show statistically significant westerly wind anomalies in the subtropical upper stratosphere (Fig. 3c), >2 m s^−1^, and in polar latitudes, extending down to the Earth's surface. This results in a stronger polar night jet throughout the depth of the polar stratosphere (60–80° N, 200–3 hPa) of >4 m s^−1^ at its maximum. As the signal propagates down to the troposphere, it slightly moves equatorward (50–70° N) projecting onto an AO/NAO-positive phase as revealed by easterly (westerly) anomalies centred at 30° N (60° N). The NO_SOL experiment shows a very similar—although weaker—zonal mean zonal wind response in the Northern Hemisphere troposphere (Fig. 3d), as shown by the easterly (westerly) wind anomalies at 30° N (55° N), consistent with the AO/NAO-positive phase. The statistically significant westerlies extend up to the lower/middle stratosphere (∼40 hPa) but easterlies are found at higher levels up to the mesosphere (above 1 hPa). Strong westerly wind anomalies are also confined to the subtropical stratopause region (Fig. 3d, 30° N, 3 hPa). Thus, in the absence of 11-year solar cycle forcing, the quasi-decadal tropospheric/lower stratospheric zonal wind is certainly linked to the AO/NAO variability, but is not related to the middle and upper stratosphere as it is in the case when solar forcing variability is included.

**Figure 4:**
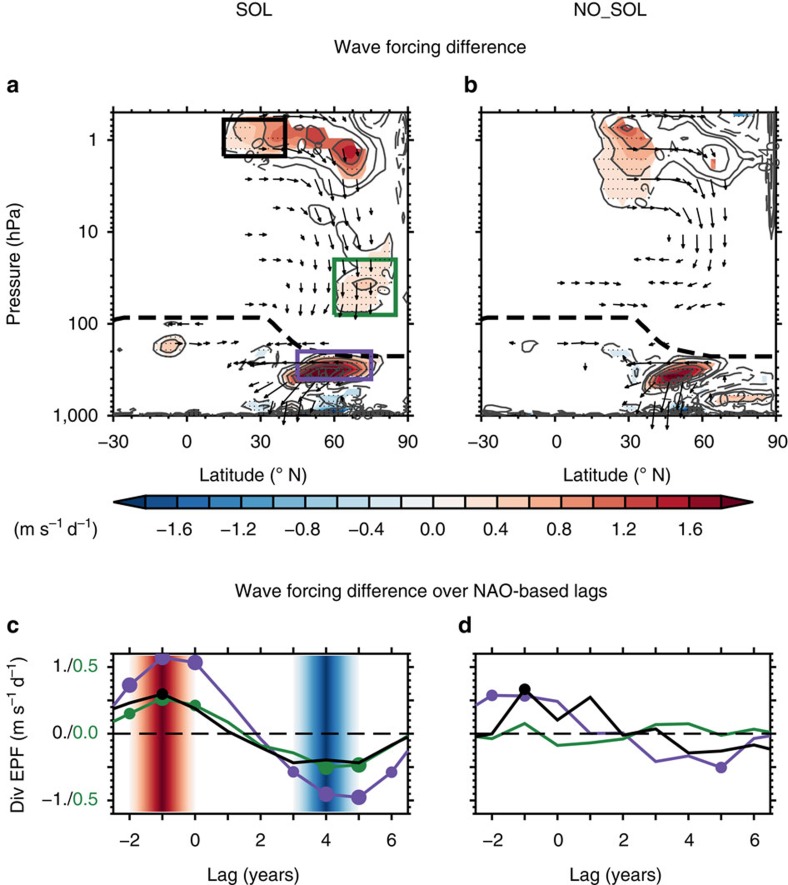
Planetary wave–mean flow interactions associated with NAO quasi-decadal variability and influence of solar cycle forcing. (**a**,**b**) Latitude-height cross-sections of NAO-based composite differences at lag −1 of the EP flux vector (arrows; scaled with the square root of the pressure) and its divergence (contours) for the SOL (**a**) and the NO_SOL (**b**) experiments. The zero contour of the EP flux divergence has been omitted for clarity. The thick dashed line displays the height of the climatological cold-point tropopause. Significance levels are indicated by shaded contours (90%) and dots (95%). (**c**,**d**) NAO-based lagged composites of the mean EP flux divergence in the subtropical stratopause (black), polar vortex edge (green) and the mid-latitude upper troposphere (violet) for the SOL (**c**) and the NO_SOL (**d**) experiment. The regions where NAO-based lagged composites (**c**,**d**) are calculated are marked by the boxes with the corresponding colours in **a**. Significance levels are indicated by small (95%) and big (99%) dots. Red and blue stripes indicate approximate lags of solar maximum and minimum phases, respectively. All calculations are shown for DJF.

Planetary wave propagation and wave–mean flow interaction anomalies are examined using NAO-based composite differences of the Eliassen–Palm flux (EPF) vector and its divergence^32^ (Fig. 4a,b). We recall that an anomalous divergence of EPF (or positive anomaly) leads to a reduced wave drag and thus a relative strengthening of westerlies. In both experiments, anomalous poleward wave refraction is observed in the vicinity of the subtropical stratopause region, leading to a statistically significant positive EPF divergence (Fig. 4a,b). This statistically significant anomaly expands poleward to 70° N in the ‘SOL' experiment (Fig. 4a), consistent with westerly wind anomalies in the upper high-latitude stratosphere (Fig. 3c). In the middle polar stratosphere (60–80° N, 100–10 hPa), the strengthening of the polar night jet (Fig. 3c) is consistent with a reduced upward planetary wave propagation and the associated westerly forcing anomaly (that is, positive EPF divergence; Fig. 4a). Note that for the SOL experiment, the zonal mean zonal wind and EPF divergence winter signals in the stratosphere associated with solar maximum minus minimum at lag −1 year of the NAO-based composites (Figs 3c and 4a) compare favourably with those obtained by Ineson *et al.*^11^ (see Fig. 4 in ref. 11; signs are inverted as they examine solar minimum minus maximum). Conversely, no statistically significant signal is observed for the NO_SOL experiment in the polar night jet region (Fig. 4b). In the upper mid-latitude troposphere, both experiments exhibit a strong EPF divergence positive anomaly (35–60° N; 400–200 hPa) associated with the positive AO/NAO signal. This signal is further amplified and expands poleward and upward when solar forcing variability is included (Fig. 4a).

The annual evolution of the December to February NAO-based lagged composite differences of the mean wave forcing anomalies in the three key regions previously identified (see boxes in Fig. 4a) shows a clear statistically significant and continuous quasi-decadal oscillation in the SOL experiment (Fig. 4c), which maximizes (minimizes) at solar maximum (minimum). Notice that the continuous interannual time evolution of the zonal mean zonal wind and wave–mean flow interaction anomalies is also visible on lagged latitude-pressure cross-sections (Supplementary Figs 7 and 8). For the NO_SOL experiment (Fig. 4d), wave forcing anomalies display a statistically significant quasi-decadal oscillation in the mid-latitude upper troposphere only, corresponding to the AO/NAO variability. The amplitude of the signal is also two times lower than in the SOL experiment. The comparison of stratospheric dynamical signals in both experiments shows that including the 11-year solar cycle leads to a stratosphere ‘top–down' influence on the tropospheric circulation that could explain the synchronization of the NAO at quasi-decadal time scales.

## Discussion

Recent analyses of the Northern Annular Mode/NAO variability conducted in the framework of the fifth phase of the Coupled Model Intercomparison Project^33^ (CMIP5) revealed no significant response to the 11-year solar cycle by examining the average over 37 models participating^34^. Mitchell *et al.*^24^ repeated a similar analysis by grouping models according to their lid height and by examining the responses at different lags. Although they found evidence that high-top models (with a lid height of at least 0.1 hPa or ∼65 km) better simulate the observed solar/NAO relationship than low-top models, the magnitude of the signals remains weak and the agreement between models is relatively low. They further noticed that the most promising solar signals seem to be obtained with models, which have a high spectral resolution and a well-resolved stratosphere including the equatorial Quasi Biennial Oscillation and interactive chemistry.

In this study, we propose a new and testable synchronization mechanism that combines air–sea interaction processes^18^ and solar-induced stratospheric dynamics modulation^11^,^13^ to simulate the observed solar influences on North Atlantic climate. Given the quasi-oscillatory behaviour of the solar cycle and that 4% of the total NAO variance can be explained by the solar variability in our SOL experiment, we believe that this mechanism could potentially improve decadal predictions. Although this contribution is relatively small regarding the NAO total variance, it represents a significant increment to other sources of predictable decadal variability^35^. A rigorous representation of the impact of ultraviolet forcing on the atmospheric thermal structure is obtained using a radiative transfer module, which adequately resolves spectral solar variability^36^,^37^ and by including effects of solar-induced ozone variations^38^. The latter can be achieved by (i) prescribing the ozone changes or (ii) interactively calculating the ozone chemistry as in our model configuration. While the first option offers the opportunity of significantly reducing the computational costs, the second option remains the most accurate to simulate ozone feedbacks on atmospheric dynamics and radiation^39^, which may be important for the representation of the solar signal^40^. More modelling sensitivity studies should be conducted to assess the relevance of implementing interactive ozone chemistry.

Finally, we note that the conservative solar spectral irradiance model used in our experiments (NRLSSI, Naval Research Laboratory Solar Spectral Irradiance) represents the lower limit in the magnitude of SSI solar cycle variation among all models and measurements available^41^. This could explain the slightly weaker upper stratosphere solar temperature signal in our experiments compared with reanalyses^31^. In this context, it is crucial to assess the best estimate of solar spectral irradiance variability to reduce model uncertainty regarding the natural climate variability.

## Methods

### Model description

We use the Community Earth System Model (CESM), developed at the National Center for Atmospheric Research (NCAR)^26^. CESM is a state of the art fully coupled model, which includes interactive ocean (POP), land (CLM) and sea ice (CICE) components, and an atmospheric component with interactive chemistry (WACCM3.5). WACCM3.5 has a horizontal resolution of 2.5° × 1.9° (longitude × latitude) and 66 vertical levels which extend from the Earth's surface up to 140 km. WACCM3.5 includes spectrally resolved solar variability from the NRLSSI data set^42^. For all experiments, the equatorial stratospheric winds are relaxed toward observed winds in exactly the same way to obtain a realistic time-varying Quasi-Biennial Oscillation^43^. The POP ocean module has a tripolar horizontal grid of 1° × 1° and 60 depth levels.

### Experimental design

Both multi-decadal experiments span 145 years running from January 1955 to December 2099. For each experiment, the anthropogenic forcings are excluded by keeping greenhouse gases and ozone depleting substances constant at 1960s levels to focus exclusively on natural climate variability. The SOL experiment is forced by daily observed spectrally resolved solar irradiance from 1955 to 2009 and by repeating twice the four last solar cycles from 2010 to 2099. The NO_SOL experiment is forced with the average of the solar irradiance time series previously defined. Both experiments include radiative forcing from volcanic eruptions over 1955–2000, including the major eruptions of Agung (1963), El Chichòn (1982) and Mount Pinatubo (1991).

### NAO index definition

The NAO refers to an oscillation of atmospheric mass between the Arctic and the subtropical Atlantic, which corresponds to the most important mode of variability in the Northern Hemisphere atmospheric circulation^44^. The NAO index is usually derived either from the difference in surface pressure anomalies between various northern and southern stations (typically located on Iceland and Azores, respectively) or from the principal component (PC) time series of the leading eigenvector of the deseasonalized SLP about the North Atlantic area. The principle of the latter—also called empirical orthogonal function (EOF) analysis—is to find the orthogonal functions, which best characterize the time series covariance of all points of a given spatial grid. The advantage of this approach is that the index gives a more optimal representation of the full NAO spatial pattern^45^.

We used the PC approach to derive NAO indices in the SOL and NO_SOL experiments and the NCEP/NCAR reanalysis. The NAO spatial pattern is defined by the leading EOF^46^ of the deseasonalized SLP over the Atlantic sector (20–80 N, 90 W–40 E) in winter season. The leading EOFs for both the model and reanalysis describe very similar large scale seesaw patterns, characterized by low (high)-pressure anomaly in polar (subtropical) region (Supplementary Figs 4a,b and 5a). This NAO mode calculated in CESM-WACCM accounts for >36% of the deseasonalized SLP variance in the Atlantic sector, in good agreement with reanalysis calculations where the explained variance reaches 40%. The leading PC corresponds to the time series of the NAO index (Supplementary Figs 4c,d and 5b). Positive (negative) NAO phase thus indicates a strengthening (weakening) of the low polar and high subtropical anomalies.

### Composite definitions

Two types of composite analyses are used in this study: (i) 11-year solar cycle-based composites to examine the solar signal in the SOL experiment and (ii) NAO-based composites to compare the SOL and NO_SOL experiments with each other. Solar cycle-based composites are defined according to the annual mean F10.7 cm radio flux, which is a good proxy for solar ultravilolet activity. After identifying solar maximum and minimum central (or peak) years for each cycle (13 in total), the two surrounding years are selected such that per cycle, each solar maximum and minimum is defined by 3 years (Supplementary Fig. 1). NAO-based composites indices are defined using the same method as for the solar cycle-based ones, except that quasi-decadal band-pass filtered NAO index time series are used instead of F10.7 index (Fig. 2). Note that the use of this composite method avoids data filtering. Lag composites are obtained by shifting all indices by a chosen lag in years.

### Statistical significance analysis

Given the high degree of serial correlation in the low-pass filtered time series, the significance of correlation between filtered NAO and F10.7 indices were assessed using a nonparametric random phase test^27^. This method preserves the spectrum and auto-correlation of the original data. In practice, we generate 1,000 synthetic random filtered NAO time series having the same power spectrum as the original one and we correlate each against the original F10.7 time series. The 1,000 correlation coefficients are used to construct a probability distribution of correlations. Regarding the composites, the significance level is estimated using a bootstrapping technique with replacement. The procedure is to select two random subsets from the original time series with the lengths equal to the two original composite subsamples. This procedure is repeated 1,000 times and a distribution of the differences is constructed. Finally, correlations and composite distributions are used to determine the likelihood of the derived signals arising by chance. One-tailed tests are used.

### Code availability

The source code of the Community Earth System Model version 1.0 (CESM 1.0) used in this study is publicly distributed and can be obtained after registration at http://www.cesm.ucar.edu/models/cesm1.0/. The algorithm used to perform EOF analysis has been written by Mark Baldwin in Interactive Data Language (IDL) and is available at http://people.nwra.com/resumes/baldwin/eofcalc.pro.

## Additional information

**How to cite this article:** Thiéblemont, R. *et al.* **S**olar forcing synchronizes decadal North Atlantic climate variability. *Nat. Commun.* 6:8268 doi: 10.1038/ncomms9268 (2015).

## Supplementary Material

Supplementary Information Supplementary Figures 1-8
