# Supplementary material for: Solar forcing synchronizes decadal North Atlantic climate variability
Source: Nat Commun. 2015 Sep 15;6:8268. doi: 10.1038/ncomms9268 (PMC4579852; doi:10.1038/ncomms9268)
Supplement: Supplementary Information — Supplementary Figures 1-8 [file ncomms9268-s1.pdf]

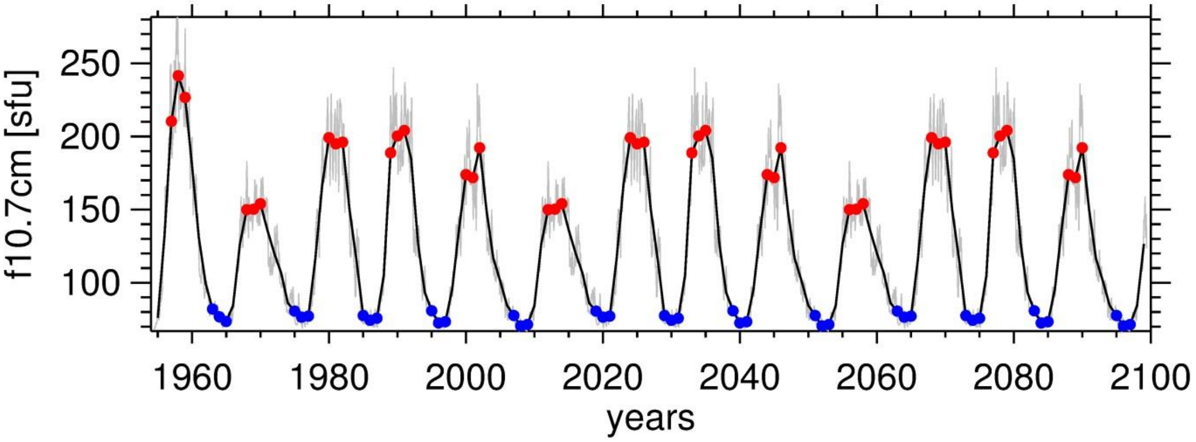

Supplementary Figure 1 | Solar cycle-based composite definition. Monthly (thin grey line) and annual (thick black line) means of the solar radio flux at 10.7 cm in solar flux units for the SOL experiment (1 sfu = $10^{-22}Wm^{-2}$ Hz^{-1}) . Red (blue) dots indicate the solar maximum (minimum) indices used for Solar cycle-based composite at lag 0.

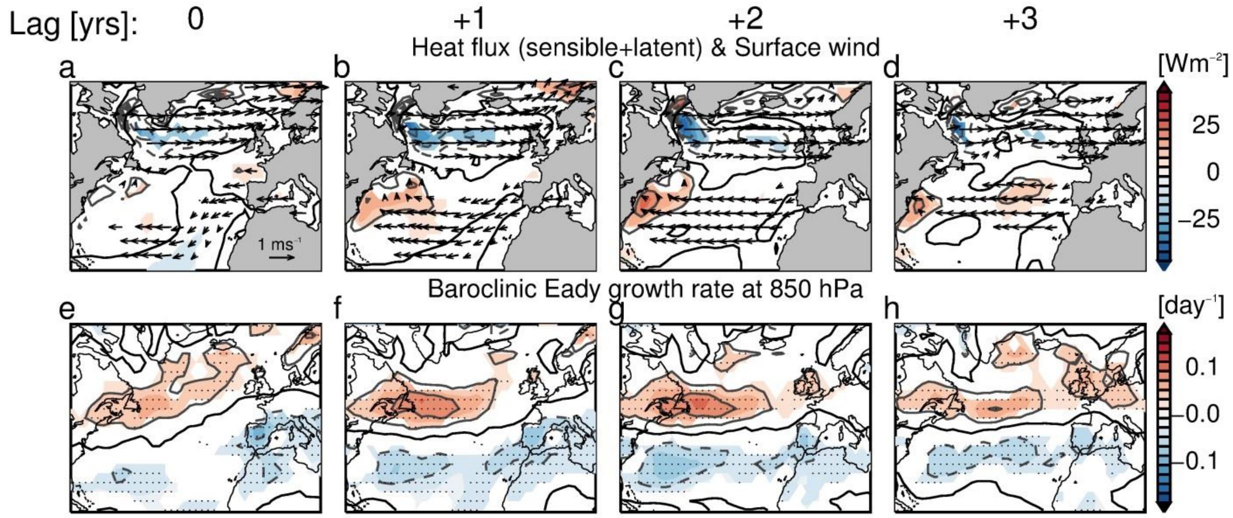

Supplementary Figure 2 | Atlantic ocean-atmosphere interactions at quasi-decadal timescales. Solar cycle-based lagged composite differences in DJF for the (**a-d**) turbulent heat flux (sensible + latent, contours) and surface wind anomalies (vectors), and (**e-h**) baroclinic Eady growth rate at 850 hPa. Significance levels are indicated by shaded contours (90%) and dots (95%). Each map shows the North Atlantic region only (80°W-20°E/20°N-70°N). Positive and negatives anomalies of heat fluxes indicate ocean heat gain and loss, respectively. (**a-d**) The SST cooling at high (low) latitudes can be explained by a strengthening of westerlies (trade winds), associated with the positive phase of the NAO, which enhances turbulent heat fluxes from the ocean to the atmosphere. The SST warming at mid-latitudes arises from a weakening of the climatological westerlies which weaken the upward heat fluxes. (**e-h**) Persistence of the tripole has a positive feedback on the NAO by enhancing the surface temperature gradient and baroclinicity through turbulent fluxes.

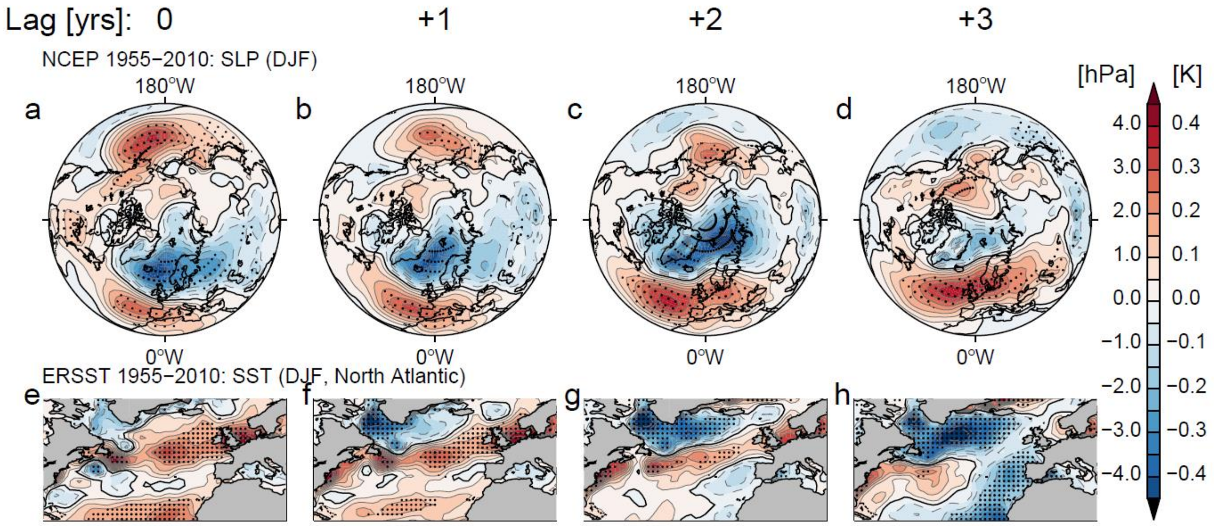

Supplementary Figure 3 | Solar signal at the surface in reanalysis for the period 1955-2010. As Fig. 1 but for the (a) SLP from NCEP/NCAR reanalysis and (b) SST from NOAA ERSST v3b. Dotted regions are significant at the 90% level.

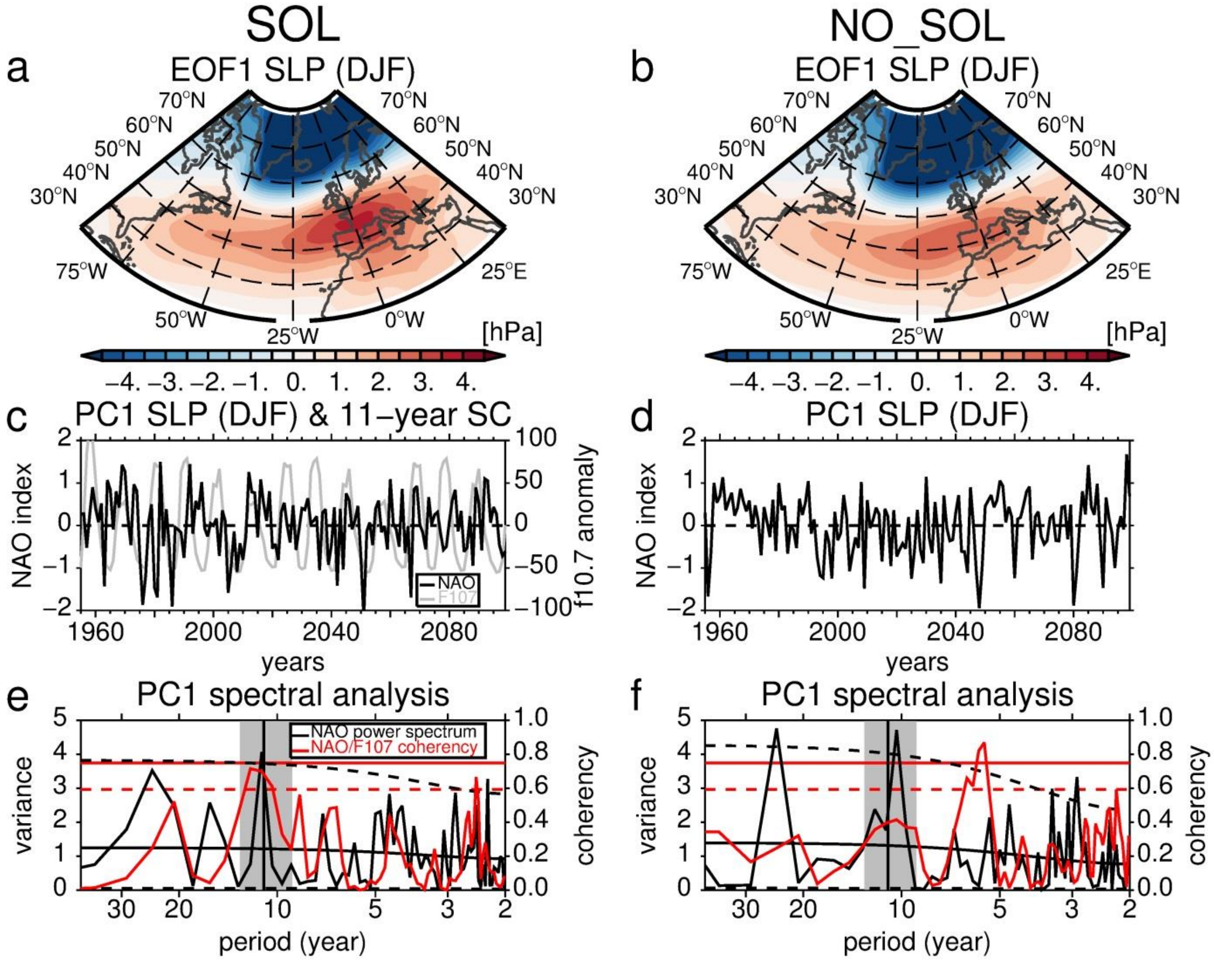

Supplementary Figure 4 | NAO index spectrum analysis with and without solar cycle forcing. (**a**) Leading Empirical Orthogonal Function of the deseasonalized sea level pressure (in hPa) in DJF for the SOL (36.0% variance explained) and (**b**) NO_SOL (36.8% variance explained) experiments. (**c,d**) NAO indices (black line) calculated as the first Principal Component derived from (**a,b**, respectively). The annual mean f10.7 cm anomaly (grey line) is superimposed for the SOL experiment panel (**c**). (**e,f**) NAO index multi taper method power spectra (black line) and spectral coherency with f10.7 for the SOL (**e**) and NO_SOL (**f**) experiments. Black dashed lines indicate the 5% (lower) and 95% (upper) confidence levels for power spectra. Solid (dashed) red line indicates the 95% (99%) confidence levels for coherency. The grey stripe between 9 and 13 years indicates the range of the bandpass filter used to derive quasi-decadal NAO-based composites. Notice that in the SOL experiment, the quasi-decadal peak is tighter than for the NO_SOL experiment and coincides exactly with a period of 11 years (vertical black line).

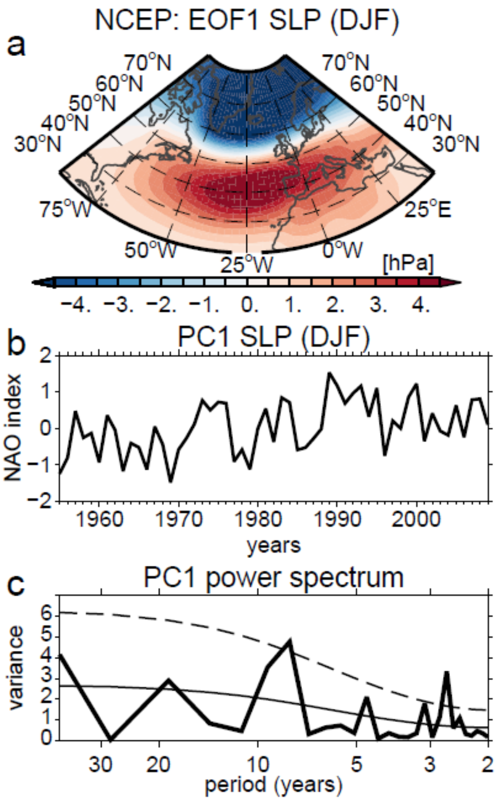

Supplementary Figure 5 | NAO index spectrum analysis in NCEP/NCAR reanalysis. (**a**) Leading Empirical Orthogonal Function of the deseasonalized sea level pressure (in hPa) in DJF for NCEP/NCAR reanalysis from 1955 to 2010 (40.0% variance explained). (**b**) NAO index calculated as the first Principal Component derived from (**a**). (**c**) NAO index multi taper method power spectra. Black dashed lines indicate the 5% (lower) and 95% (upper) confidence levels for power spectra

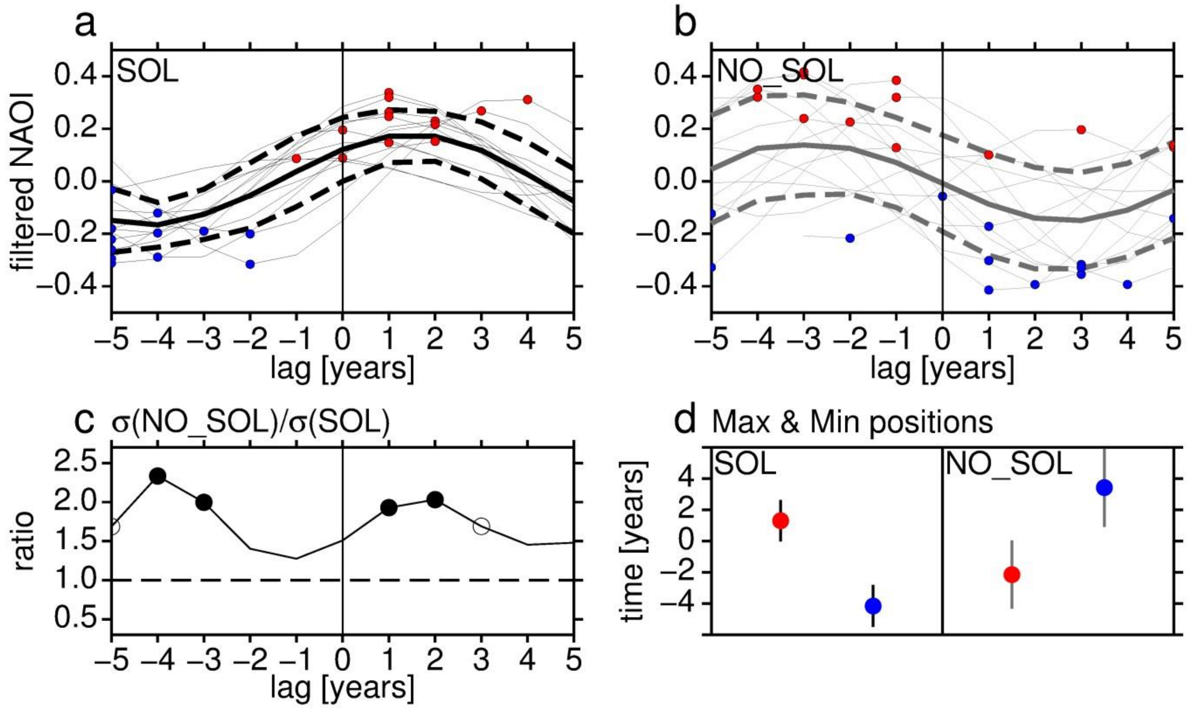

Supplementary Figure 6 | Comparison of the SOL and NO_SOL NAO quasi-decadal fluctuations. Superimposed quasi-decadal band-pass filtered SOL (**a**) and NO_SOL (**b**) NAO time series as a function of a common reference taken as the solar maximum (vertical lines on Fig. 2a). The mean and standard deviation are indicated by the thick solid and dashed lines, respectively. (**c**) Ratio of the standard deviations of NO_SOL NAO to SOL NAO indices at each lag. The filled (empty) dots indicate that the variances are significantly different at the 95% (90%) level according to a Fisher test. (**d**) Mean temporal position (in years) of the NAO maxima (red dots) and minima (blue dots) relative to the same time reference (solar maximum; year 0) for the SOL and NO_SOL experiments. Vertical lines show the standard error.

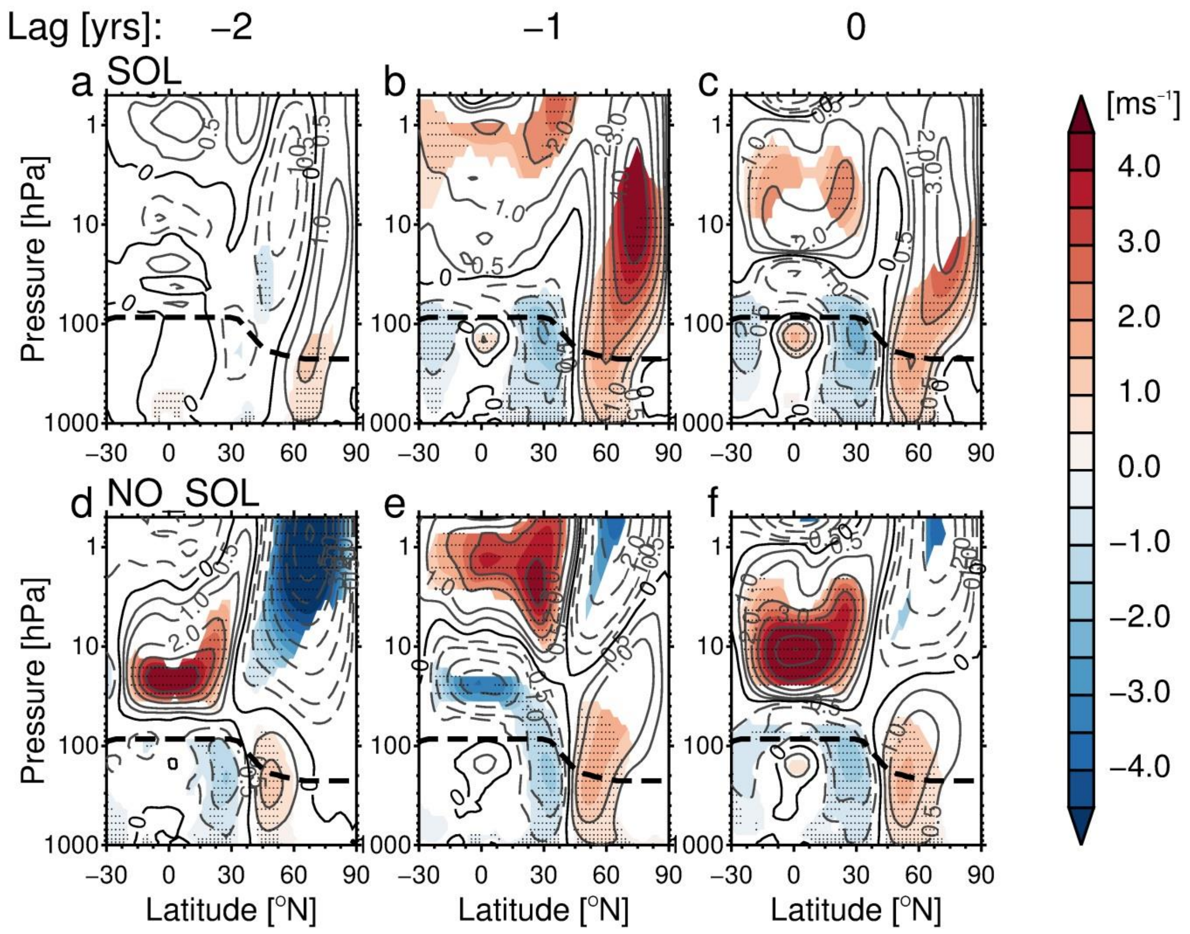

Supplementary Figure 7 | Zonal mean zonal wind response associated with NAO quasi-decadal oscillation, with and without solar forcing. Latitude-height cross sections from 30°S to 90°N and 1000 hPa to 0.5 hPa of NAO-based composite differences of the zonal mean zonal wind (in ms^{-1}) at lags -2 (**a,d**), -1 (**b,e**) and 0 years (**c,f**) for the SOL (**a-c**) and NO_SOL (**d-f**) experiments. Significance levels are indicated by shaded contours (90%) and dots (95%). All calculations are shown for DJF.

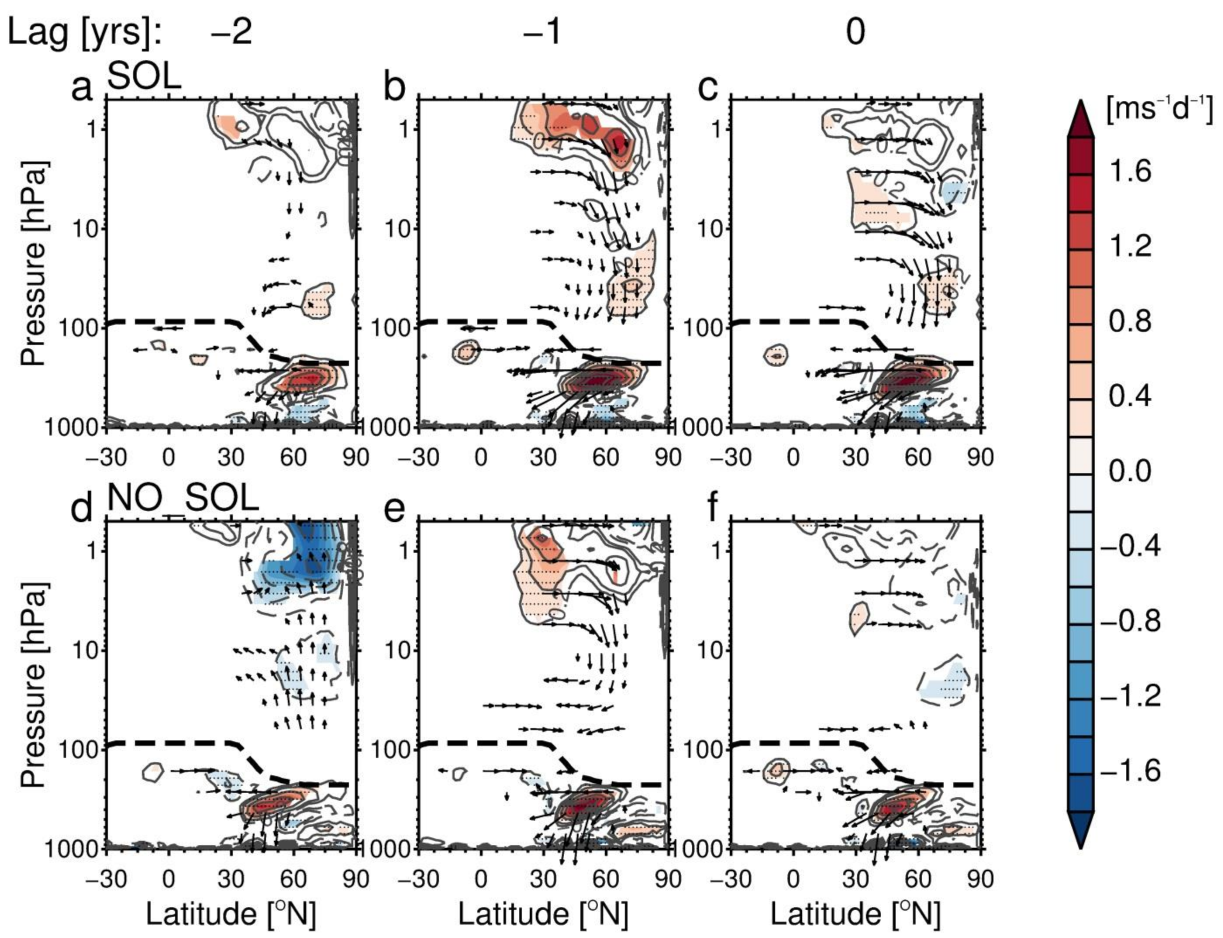

Supplementary Figure 8 | Wave and wave-mean flow interaction response associated with NAO quasi-decadal oscillation, with and without solar forcing. Same as Fig. S6 but for the Eliassen-Palm flux vector (arrows) and its divergence (contour, in $ms^{-1}d^{-1}$).
